# Bidimensional SnSe_2_—Mesoporous Ordered Titania Heterostructures for Photocatalytically Activated Anti-Fingerprint Optically Transparent Layers

**DOI:** 10.3390/nano13081406

**Published:** 2023-04-19

**Authors:** Jessica De Santis, Valentina Paolucci, Luigi Stagi, Davide Carboni, Luca Malfatti, Carlo Cantalini, Plinio Innocenzi

**Affiliations:** 1 Department of Industrial and Information Engineering and Economics, 67100 L’Aquila, Italy; jessica.desantis@graduate.univaq.it (J.D.S.); valentina.paolucci2@univaq.it (V.P.); 2 National Interuniversity Consortium of Materials Science and Technology (INSTM), 50121 Florence, Italy; dcarboni@uniss.it (D.C.); lucamalfatti@uniss.it (L.M.); 3 Laboratory of Materials Science and Nanotechnology (LMNT), Department of Biomedical Sciences, CR-INSTM, University of Sassari, 07100 Sassari, Italy; lstagi@uniss.it; 4 Department of Chemistry, College of Science, United Arab Emirates University, Al Ain 15551, United Arab Emirates

**Keywords:** SnSe_2_, 2D materials, titania, photocatalysis, anti-fingerprint

## Abstract

The design of functional coatings for touchscreens and haptic interfaces is of paramount importance for smartphones, tablets, and computers. Among the functional properties, the ability to suppress or eliminate fingerprints from specific surfaces is one of the most critical. We produced photoactivated anti-fingerprint coatings by embedding 2D-SnSe_2_ nanoflakes in ordered mesoporous titania thin films. The SnSe_2_ nanostructures were produced by solvent-assisted sonication employing 1-Methyl-2-pyrrolidinone. The combination of SnSe_2_ and nanocrystalline anatase titania enables the formation of photoactivated heterostructures with an enhanced ability to remove fingerprints from their surface. These results were achieved through careful design of the heterostructure and controlled processing of the films by liquid phase deposition. The self-assembly process is unaffected by the addition of SnSe_2_, and the titania mesoporous films keep their three-dimensional pore organization. The coating layers show high optical transparency and a homogeneous distribution of SnSe_2_ within the matrix. An evaluation of photocatalytic activity was performed by observing the degradation of stearic acid and Rhodamine B layers deposited on the photoactive films as a function of radiation exposure time. FTIR and UV-Vis spectroscopies were used for the photodegradation tests. Additionally, infrared imaging was employed to assess the anti-fingerprinting property. The photodegradation process, following pseudo-first-order kinetics, shows a tremendous improvement over bare mesoporous titania films. Furthermore, exposure of the films to sunlight and UV light completely removes the fingerprints, opening the route to several self-cleaning applications.

## 1. Introduction

The photocatalytic activity of anatase nanocrystalline titania is a well-known phenomenon with manifold applications, such as energy conversion, photonics, and photoinduced antimicrobial activity [1]. In particular, the technologies based on thin films are successful examples of applications of the photocatalytic properties of titania [2]. A thin layer of transparent TiO_2_ deposited on window glasses or lenses enables the so-called self-cleaning effect [3], which causes the decomposition of the organic compounds on the glass surface thanks to the sunlight absorption [4]. Several products based on this phenomenon are already on the market; however, their ability to completely decompose solid pollutants on lenses and glass facades has not yet fully developed, and the main benefit is the need of less-frequent cleanings of the surfaces. On the other hand, efficient self-cleaning materials can also be applied as anti-fingerprint and anti-smudge surfaces [5,6] which are highly requested in many applications, such as in touchscreens for smart phones [7], photovoltaic devices, and anti-smudge eyeglasses [8]. At the moment, the technological challenge is still the fabrication of thin photoactive layers that do not modify some key properties of the surface, such as optical transparency and reflectance. Nonetheless, the most common coatings developed for this purpose are fluorinated [9] or polymeric films [10], which have adhesion and/or durability problems on glass surfaces.

The use of an ordered mesoporous titania layer instead of a dense thin film provides a strong improvement of the photocatalytic self-cleaning effect, which is up to four times faster than that measured on the nonporous coating [11,12]. Further developments are now envisaged thanks to the possibility of fabricating titania-based heterostructures in mesoporous layers. The potential of 2D layered materials, especially metal chalcogenides, has been investigated across many fields, such as photocatalysis [13,14,15], optoelectronics [16], and sensing [17]. The formation of heterostructures using 2D materials has been confirmed as a promising strategy to enhance the photocatalytic response in thin transparent mesoporous ordered titania films [18,19]. Several 2D layered materials, depending on their bandgap, can be combined with nanocrystalline titania to yield photoactive heterostructures with a tailored light-harvesting capacity. The fabrication of optically transparent thin films integrating 2D materials is a challenging goal. For several applications, as we previously underlined, the photoactive layer must preserve the transparency. This is a mandatory property for photovoltaic devices, self-cleaning coatings on window glass, or touch screens. Titania-based heterostructures that employ metal nanoparticles (i.e., gold or silver) also work well to improve the photocatalytic performances, but, regrettably, they reduce the overall transmittance by coloring the films. In previous works, we successfully formed photoactive heterostructures by fabricating mesoporous titania nanocomposites embedding 2D materials [20,21,22]. Graphene, boron nitride, and WS_2_ materials have been incorporated into mesoporous titania thin films. By adopting a one-pot synthesis, the method we devised enables the fabrication of transparent films that include 2D components as the dispersed phase. 

Here we report the synthesis of SnSe_2_-loaded mesoporous ordered TiO_2_ films through liquid phase deposition. The choice of SnSe_2_ is based on its intrinsic ability to trigger surface chemical reactions, as in the case of chemical sensors [23], and its narrow bandgap that ensures a broad absorption in the whole solar spectrum. Moreover, theoretical studies have shown that SnSe_2_ can efficiently act as co-catalyst in water-splitting reactions, as it promotes charge separation and provides trapping and reduction sites for protons. SnSe_2_, however, also exhibits a fast electron recombination and has a low conduction band (CB) level (−0.16 eV vs. the normal nitrogen electrode (NHE)); therefore, it has to be coupled with other semiconductors, such as TiO_2_, to reduce the recombination of charge carriers and increase the rate of radical formation [24]. The formation of SnSe_2_/TiO_2_ heterostructures in thin mesoporous films allows us to improve the self-cleaning capability while keeping the level of transparency the same. Achieving such a condition, however, requires a careful design of the coating to avoid SnSe_2_ aggregation. Furthermore, the as-deposited titania films, after dip- or spin-coating, must be thermally annealed to form the anatase crystallites. The annealing process, however, has to be finely tuned to avoid oxidation of the SnSe_2_. The addition of few-layer SnSe_2_ into the titania precursor sol allowed us to achieve great control over the final chemo-physical properties of the nanocomposites with a remarkable improvement of the photocatalytic performance. The resulting heterostructure was used to fabricate highly efficient self-cleaning and anti-fingerprint optically transparent thin films.

## 2. Materials and Methods

### 2.1. Materials

Powder SnSe_2_ crystals (Ossila Ltd—Sheffield, UK, CAS 20770-09-6), 1-Methyl-2-pyrrolidinone (NMP), (Sigma-Aldrich, Milano, Italy, CAS 872-50-4), titanium(IV) chloride (TiCl_4_) (Sigma-Aldrich, Milano, Italy, CAS 7550-45-0), ethanol (EtOH), (Sigma-Aldrich, Milano, Italy, CAS 64-17-5), Pluronic F-127 (~12,600 g·mol^−1^, Sigma-Aldrich, Milano, Italy CAS 9003-11-6), stearic acid (Sigma-Aldrich, Milano, Italy, CAS 57-11-4), Rhodamine B (RhB), (Sigma-Aldrich, Milano, Italy, CAS 81-88-9), and deionized water were used as received, without further purification. (100) oriented, P-type/Boron-doped silicon wafers and synthetic fused silica slides (Suprasil^®^, Hanau, Germany) were employed as the substrates.

### 2.2. SnSe_2_-Nanomaterial Synthesis

A total of 0.020 g of SnSe_2_ powder was dispersed into 40 mL of NMP used as solvent and sonicated by a bath sonicator for 6 h (Elmasonic P, working at 37 kHz) in a thermostat bath to prevent temperature rise (T 25 °C). After sonication, the suspension was centrifuged at 500 rpm for 20 min to precipitate the non-exfoliated material. The supernatant was then filtered (nitrocellulose filter with 0.45 µm pore size) and subsequently washed several times with ethanol to remove the residual NMP solvent. Finally, the filtered SnSe_2_ powder was dried at 60 °C overnight. For microstructural characterization of the exfoliated flakes, SnSe_2_ nanosheets were redispersed in ethanol and spin-coated on silicon substrates.

### 2.3. Synthesis of Mesoporous Ordered SnSe_2_-TiO_2_ Heterostructures

An evaporation-induced self-assembly method was used to synthesize the mesoporous films. Initially, a titania–stock solution (Ti-stk) was prepared by adding 4.4 mL of TiCl_4_ in 40 mL of EtOH. Then 248 mg of Pluronic F-127 was dissolved in 5.07 mL of ethanol, and after 15 min of stirring, 4.23 mL of Ti-stk and 0.687 mL of deionized water were dropped into the mixture, following this sequence. The final solution (10 mL) was stirred for 2 h to obtain the precursor sol. Then 0.02 g of powdered SnSe_2_ nanoflakes was added to the precursor sol.

Silicon wafers and silica glass were used as substrates for the deposition of thin films via dip-coating. Before the film deposition, the silicon wafers were rinsed with ethanol and dried with compressed air. The fused silica glass slides were cleaned with an aqueous solution containing an ionic detergent, rinsed with acetone, washed with ethanol, and then dried with compressed air.

**Figure 1:**
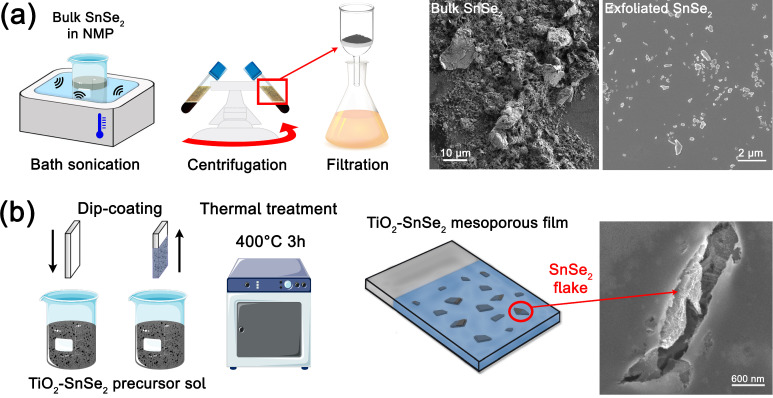
The schematic process of (**a**) exfoliation process of SnSe_2_ powder and (**b**) preparation of mesoporous ordered titania films containing dispersed nanoflakes of SnSe_2_.

The substrates were immersed in the SnSe_2_-titania sol for 30 s and withdrawn at a 10 cm min^−1^ rate. During the process, the relative humidity was kept below 25% by a dried air stream within the deposition chamber. Lastly, the obtained films were thermally annealed in air at 400 °C for 3 h in an oven; the samples were inserted directly into the oven at 400 °C. The overall process is illustrated in Figure 1.

In the article, the mesoporous ordered films are indicated as m-TiO_2_, and the meso-porous ordered films containing the SnSe_2_ nano flakes as m-TiO_2_-SnSe_2_.

### 2.4. Characterizations of the Materials

The microstructure of bidimensional SnSe_2_ flakes and mesoporous films was investigated by scanning electron microscopy (SEM) (Gemini FESEM 500), working at an accelerating voltage of 2 kV. An element analysis was performed by using energy-dispersive X-ray spectroscopy (EDS).

Transmission electron microscopy (TEM) with an accelerating voltage of 100 kV and 0.34 nm resolution (TEM Philips CM100) was used to investigate the morphology and mesoporosity of TiO_2_ and TiO_2_-SnSe_2_ films.

Flake thickness was estimated by Atomic Force Microscopy (AFM); the measures were performed in air by using a NT-MDT Ntegra AFM operating in tapping mode.

The N_2_ sorption isotherms of the dried and 400 °C annealed sol were obtained using a Quantachrome Nova 1200e, and the specific surface area was determined with the Brunauer-Emmett-Teller (BET) method.

X-Ray Diffraction was performed by XRD-PAN Analytical X’PERT Pro, using Cu K_α1_ radiation (λ = 1.5406 Å) with an angle step of 0.013°.

Raman spectra were acquired using a Senterra confocal Raman microscope in the 65–1555 cm^−1^ range, with a laser excitation wavelength of 532 nm, 50X objective, and nominal power of 12.5 mW.

An α-SE-Wollam spectroscopic ellipsometer with fixed-angle geometry (Complete EASE v. 4.2 software) was used to measure the refractive index, thickness, porosity, and SnSe_2_ loading of the films. Two or three component models with void and Cauchy film were used to fit the data.

Dataphysics OCA 20 was used to evaluate the contact angle. For the measure, 2 μL water droplets deposited on the film surface were employed. The final value was obtained by averaging three measurements.

Bruker infrared Vertex 70 V interferometer was used to obtain Fourier-transform infrared (FTIR) absorption spectra. The spectra were acquired in the range 400–4000 cm^−1^ (scans average of 128 and 4 cm^−1^ of resolution).

UV-Vis spectra in the 200–800 nm range were recorded by using a Nicolet Evolution 300 UV-Vis spectrometer, with an integration time of 1.5 s per step, using a fused-silica glass substrate.

Micro-FTIR imaging of fingerprints deposited on films was performed using a Thermo Fisher Nicolet iN10M spectrometer in transmission mode, equipped with an MCT-A detector (7800–650 cm^−1^ spectral range). The fingerprints were obtained using an aqueous solution of stearic acid (5 g L^−1^) to wet the fingers. The area used of the collected image was 150 × 150 μm, with a spectral resolution of 8 cm^−1^ for 128 scans; the spectra were normalized using the stearic acid band peaking at 2919 cm^−1^ versus the Si-O-Si substrate band peaking at 1111 cm^−1^.

### 2.5. Evaluation of Photocatalytic Activity of the Films

Stearic acid and Rhodamine B were chosen as target molecules to evaluate the room-temperature photocatalytic activity and anti-fingerprint property of TiO_2_ and TiO_2_-SnSe_2_ mesoporous films deposited on Si substrates.

Stearic acid was dissolved in EtOH (5.0 g L^−1^), and 100 µL of solution was deposited on the titania films by spin-coating at 1500 rpm for 30 s. The backside of the slide was cleaned with ethanol to remove residual solution. After deposition, the films were irradiated using a UV lamp (365 nm) (nominal power density of 470 μW cm^−2^ at 15 cm) at a distance of 1 cm for different illumination times, 0-15 min. FTIR spectra were recorded after each 2.5 min illumination step.

The photodegradation of stearic acid on the films was evaluated by FTIR analysis by considering the change of the vibrational modes at 2945 and 2845 cm^−1^ (-CH_2_ and -CH_3_ stretching, respectively) by averaging 128 scans with 4 cm^−1^ of resolution. The change in the corresponding area of the infrared bands as a function of irradiation time was estimated using the FTIR spectra. The integral of the areas, ranging from 2800 to 3010 cm^−1^, were used for the evaluation.

Rhodamine B (RhB) was also used as a target molecule to evaluate the photocatalytic activity of films deposited in fused silica slides. The integral of the absorption bands, ranging from 450 to 625 nm, was used to monitor the changes induced by the UV exposure.

The Rhodamine B solution for the photocatalytic test was prepared by dissolving RhB powder in EtOH (5·10^−3^ mol L^−1^); 100 µL of solution was spin-coated at 1500 rpm for 30 s on the selected substrate. The backside part of the films was cleaned with ethanol to remove the eventual presence of the residual solution. After the deposition, the films were irradiated under 365 nm, using a UV lamp for 0–50 min, and the UV-Vis spectra were re-corded immediately after illumination. To obtain a precise readout on the photodegradation kinetics of RhB, a baseline subtraction was carried out with the use of a spline fit. This procedure was employed to suppress the interference pattern produced by the thin films. Calculation of the baseline is critical because it affects the evaluation of the photocatalytic activity.

### 2.6. Evaluation of Rhodamine B Degradation in Liquid Phase

The photodegradation test of Rhodamine B in water (2.5·10^−6^ mol L^−1^) was carried out in a UV-Vis cuvette. The degradation of Rhodamine B in aqueous solution caused by UV light exposure was compared to that resulting from the addition of bidimensional SnSe_2_ flakes to the solution. For the test, a 3 mL aqueous solution and a concentration of 0.07 g L^−1^ SnSe_2_ were employed. The UV lamp (365 nm, Led Zolix instruments, M365L 420 mW, 90% power) was positioned at a distance of 3 cm. To avoid sedimentation of SnSe_2_ flakes, the cuvettes were stirred during the UV exposure. The integral of Rhodamine B absorption spectra in the range of 400–650 nm was used to evaluate the effect of UV irradiation; the data were smoothed using the Savitzky–Golay algorithm.

## 3. Results and Discussion

The synthesis of m-TiO_2_-SnSe_2_ heterostructures requires well-exfoliated SnSe_2_ nanostructures, which can be uniformly dispersed in the titania precursor sol. Among the several exfoliation processes used to obtain 2D materials, liquid-phase exfoliation (LPE) stands out due to its scalability and reproducibility. Small fragments of SnSe_2_, in the shape of flakes, were obtained by LPE. These nanostructures were characterized by combining different techniques.

### 3.1. Microstructural Characterization

**Figure 2:**
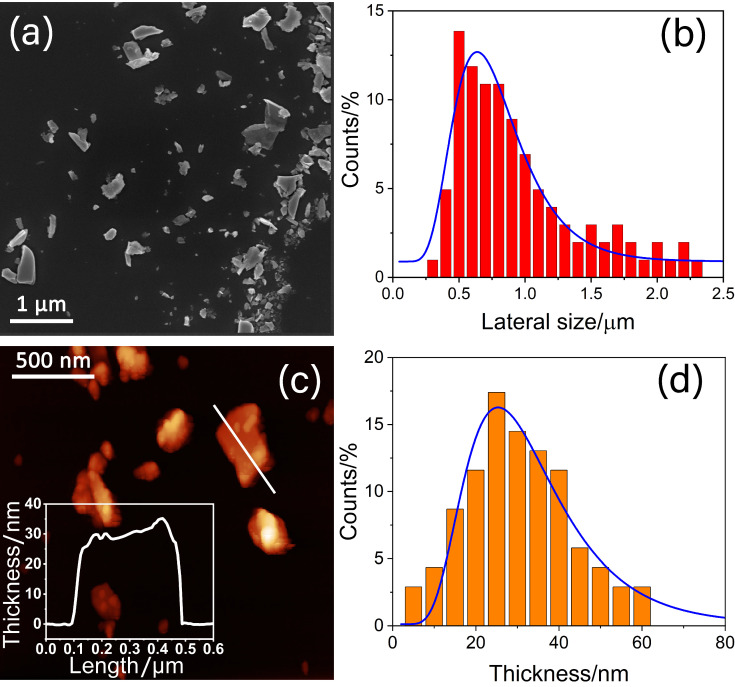
(**a**) High-resolution SEM image of representative SnSe_2_ nanoflakes. (**b**) Statistical analysis of lateral dimension calculated from SEM images. The blue curve represents the Gaussian fit. (**c**) Representative AFM image of SnSe_2_ nanoflakes. The inset shows the height profile of the flake indicated by the segment in white. (**d**) Statistical analysis of the SnSe_2_ flake thickness distribution calculated from AFM images. The blue curve is the log-normal distribution fit.

Figure 2a shows the high-resolution SEM image of representative 2D-SnSe_2_ stacked flakes exfoliated in NMP solvent. The statistical analysis (Figure 2b) displays that the flakes have an average lateral dimension of 0.738 ± 0.022 µm, similar to what reported in previous works [25]. The thickness of the flakes, as evaluated by AFM (Figure 2c), follows a log-normal distribution peaking at ∼ 30 nm (Figure 2d). Considering a theoretical thickness of a SnSe_2_ monolayer at room temperature of 1.2 nm [26], the nanoflakes consist of roughly 25 layers. The width-to-thickness ratio is 24.6, which is consistent with previous reports for bidimensional SnSe_2_ flakes synthesized in a similar manner [27,28].

**Figure 3:**
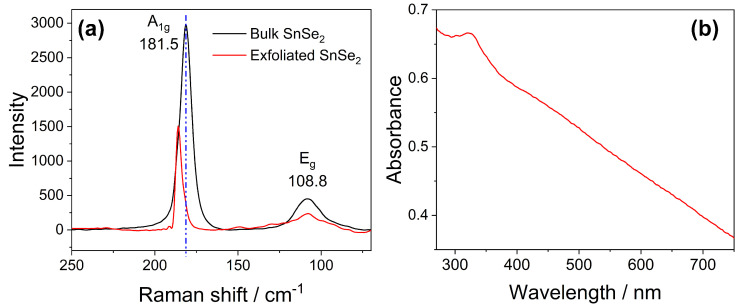
(**a**) Raman spectra of bulk SnSe_2_ (black line) and exfoliated SnSe_2_ nanoflakes (red line) (λ_ex_ = 532 nm). The blue dot line is a guide for eyes. (**b**) UV-Vis absorption spectrum SnSe_2_ flakes dispersed in ethanol (0.83 g L^−1^).

The Raman analysis was used to study the structure of bulk and exfoliated SnSe_2_ samples (Figure 3a). The SnSe_2_ bulk shows a strong band peaking at 181.5 cm^−1^ due to out-plane stretching of the A_1g_ mode and a second band of smaller intensity at 108.8 cm^−1^ assigned to the E_g_ mode of the in-plane stretching [29]. The position of the SnSe_2_ E_g_ Raman shift is indicative of the polytypes [30]: 2H-SnSe_2_ crystals have an E_g_ mode located at ~108 cm^−1^, while 1T-SnSe_2_ crystals exhibit an E_g_ mode located at ~118 cm^−1^ [31]. The observed Raman shift of the Eg peak at 108.8 cm^−1^ indicates that, in the present case, the bulk is a 2H-SnSe_2_ polytype.

Previous research has shown a correlation between the Eg band strength and the number of SnSe_2_ layers. Nevertheless, this relationship does not follow a linear trend; in fact, for flakes thicker than ten layers, the intensity does not grow any longer or even decreases due to the interference effect. [32] The ratio of A_1g_ and E_g_ band intensities is consistent with a flake thickness greater than ten layers in agreement with SEM observations. A comparison between the Raman spectra after and before exfoliation process also reveals a ~3.5 cm^−1^ redshift of the A_1g_ band. The shift is compatible with a reduced number of layers with respect to the bulk, in accordance with previous findings [24].

Figure 3b displays the UV-Vis absorption spectra of exfoliated SnSe_2_ nanoflakes in ethanol between 270 and 800 nm. The spectrum shows an absorption band at ~350 nm [33] emerging from an intense broadband absorption. The extended interval of absorption suggests that SnSe_2_-based materials can be efficiently used as a light absorber in a wide range from, UV-Vis up to the red region [34,35]. The band at 350 nm, in accordance with previous findings, is attributed to the transition from the crystal field split selenium p_xy_-like levels into tin p-like levels [36].

The film thickness was evaluated by spectroscopic ellipsometry, which allows us to also calculate the porosity and the loading percentage of SnSe_2_. The data were collected from a series of three samples prepared in the same conditions to evaluate the reproducibility. The data were fit while keeping the mean squared error (MSE) below 25. The average thickness of bare m-TiO_2_ films is ~120 nm, which is ~20 nm lower than that of the SnSe_2_-loaded samples (Table 1). The change in the sol viscosity induced by the addition of 2D nanoflakes explains the increase in thickness. The residual porosity in the m-TiO_2_ -SnSe_2_ nanocomposites is around 10% lower than that of the m-TiO_2_ films; the difference is attributed to the incorporation of ~ 7% of exfoliated SnSe_2_ nanoflakes.

**Figure 4:**
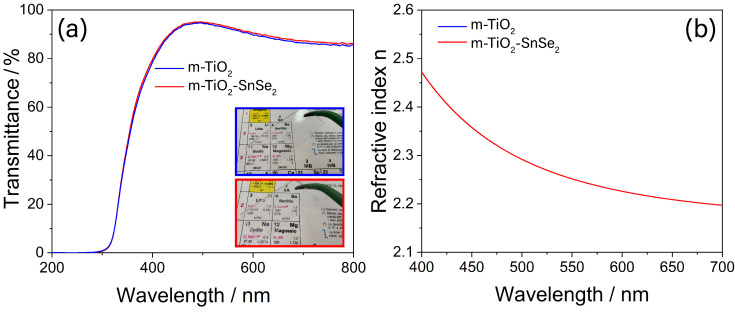
(**a**) UV-Vis spectra and refractive index (**b**) of m-TiO_2_ film (blue line) and m-TiO_2_-SnSe_2_ film (red line). The two curves overlap. The inset in (**a**) shows the snapshots of the films deposited on silica glass slides.

Figure 4a shows the transmittance spectra of m-TiO_2_ and m-TiO_2_-SnSe_2_ films. The spectra perfectly overlap with each other, with a cutoff around 350. The transmittance is higher than 85%, indicating that loading the mesoporous films with ~7% of bidimensional SnSe_2_ flakes (see Table 1) does not cause any sensible change in the transparency of the film, as shown in the insets in Figure 4a [19,37]. This result is not surprising considering the low percentage of loading and the low film thickness. The dispersion curves of the refractive index, n, also confirm that the loading process does not affect the optical properties (Figure 4b). The value of n is 2.25 for both samples at 550 nm, and this is in line with previous works that reported for anatase TiO_2_ films a refractive index in the range of 2.10–2.50 [31].

The high optical transparency is a critical requirement for most applications, such self-cleaning and anti-fingerprint layers that also need significant photocatalytic activity.

**Figure 5:**
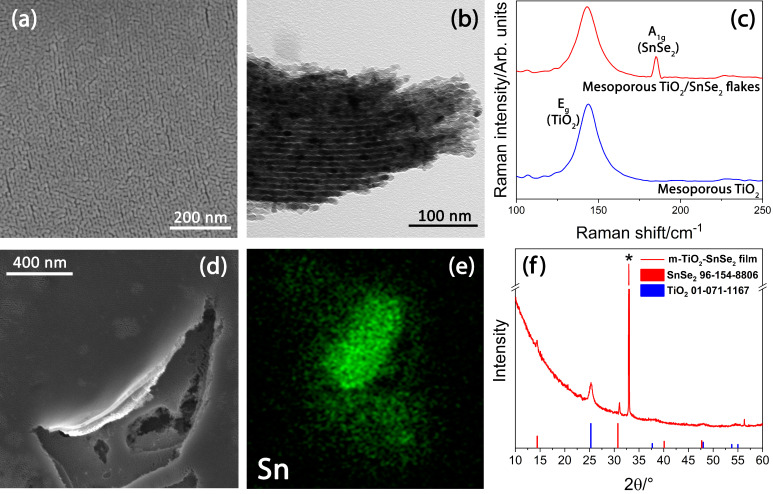
(**a**) FESEM and (**b**) TEM images of m-TiO_2_-SnSe_2_ film. (**c**) Raman comparison of m-TiO_2_ film (blue line) and m-TiO_2_-SnSe_2_ film (red line). (**d**,**e**) FESEM image and EDS analysis of SnSe_2_ flakes into m-TiO_2_ film and (**f**) GI-XRD pattern of m-TiO_2_-SnSe_2_ film. The asterisk indicates the (200) diffraction of the Si substrate.

The surface morphology of the nanocomposite films was investigated by SEM analysis (Figure 5a). The picture displays that the titania self-assembled films achieve a well-organized mesoporous structure (surface area of 112.60 m^2^/g, obtained by the BET equation fit for N_2_ gas adsorption isotherm reported in Supplementary Figure S4), and the organization is not disrupted by the presence of the SnSe_2_ flakes. These results have already been observed for mesoporous titania films embedding exfoliated graphene [19] and can be explained by considering that the mesopores size (5–6 nm) is much smaller than the lateral size of 2D-SnSe_2_ flakes (≈700 nm). The self-assembly process of the block copolymer occurs on a smaller scale with respect to the flake size and, therefore, is not affected by the addition of 2D-SnSe_2_ to the sol. On the other hand, the presence of a bidimensional material in the precursor sol favors the micelle nucleation lowering the interfacial energy through heterogeneous nucleation [38]. The mesopore organization is clearly visible from the SEM surface images and shows the typical grid-like structure, with partial merging of some pore walls on the topmost layer, in agreement with previous results [39]. The images obtained by the TEM analysis (Figure 5b) confirm the formation of spherical mesopores with a body-centered cubic ordered structure (Im3m) within the titania films [40]. The appearance of the channel-like pore arrangement is an effect generated by the transmission microscope that projects the Im3m spherical pore array in the [110] direction.

The existence of an organized mesoporosity is crucial, as the large surface increases the photocatalytic activity, while the pore organization promotes the diffusion processes [41]. The highest diffusivity within the mesoporous matrix is achieved when the pores are ordered in a body-centered cubic fashion, whereas 2D hexagonal arrays of cylindrical channels provide limited diffusion due to pore packing defects [42,43]. The presence of SnSe_2_ within the titania matrix was confirmed by the FESEM analysis of a scratched area of the film (Figure 5d). The SEM picture confirms the presence of exfoliated SnSe_2_ flakes, as also confirmed by the EDS analysis (Figure 5e), which detects Sn and Se atoms (see Supplementary Figure S2). The successful incorporation of the SnSe_2_ nanoflakes into m-TiO_2_ film is also supported by the Raman analysis (Figure 5c). The pure mesoporous titania films show a Raman mode located at 144 cm^−1^ that is the signature of the typical E_g_ vibration mode of TiO_2_ anatase phase (blue line) [44]. This mode shows the same intensity and position in the nanocomposite m-TiO_2_-SnSe_2_ films (red line). Additionally, the Raman spectra show a new mode located at ~183 cm^−1^ that is attributed to the A_1g_ mode of SnSe_2_ nanoflakes (Figure 3a) [26].

The microstructure of the m-TiO_2_-SnSe_2_ films was further assessed by GI-XRD analysis (Figure 5f). The X-ray pattern indicates the crystallization of titania in anatase, as shown by the main diffraction peak at 25.3 attributed to the (110) plane of TiO_2_ anatase phase (# JCPDS No. 01-071-1167) [45]. The average crystallite size of the TiO_2_ nanoparticles is ~15.5 nm, calculated using the Scherrer equation. Additionally, the diffraction peaks detected at 14.3 and 31.1° in 2θ h were assigned to the (001) and (101) planes of the SnSe_2_ nanostructures (# JCPDS No. 96-154-8806) [46].

The XRD data, in combination with SEM, FESEM, TEM, and Raman, allow us to obtain an overall characterization of the nanocomposite mesoporous films. After the thermal treatment at 400 °C, titania crystallizes into anatase, which is the active photocatalytic phase. At the same time, the phase transformation keeps the organization of the mesopores, while the SnSe_2_ flakes are homogeneously distributed within the matrix. It is important to underline that the introduction of the bidimensional SnSe_2_ flakes via the one-pot route does not interfere with the self-assembly process, particularly with the organization of the supramolecular template that triggers the formation of the mesophase (see Supplementary Figure S3).

Contact angle measurements were used to investigate the surface wettability of the films (see Supplementary Figure S5), since this is, in fact, an important property to be assessed for anti-fingerprint coatings. Despite the presence of loaded SnSe_2_ nanoflakes, the contact angle remains the same as that of the unloaded film (~23°) [17], indicating that the surface keeps the hydrophilic properties.

### 3.2. Photocatalytic Degradation of Rhodamine B by SnSe_2_ Nanoflakes

**Figure 6:**
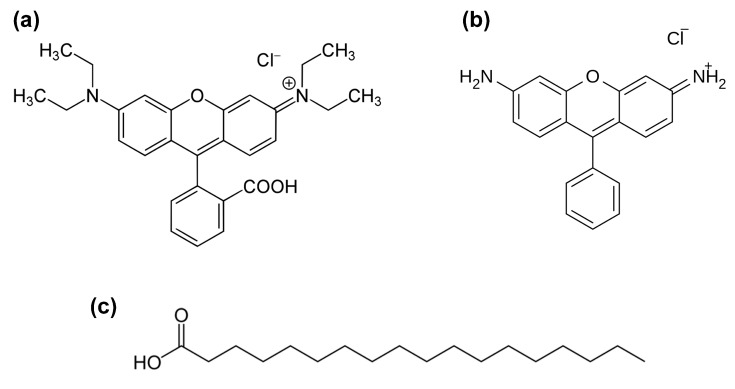
Chemical structure of Rhodamine B (**a**), Rhodamine 110 (**b**), and stearic acid (**c**).

The self-cleaning properties of the nanocomposites films were tested by following the photocatalytic degradation under UV irradiation of two different molecules, Rhodamine B (RhB) and stearic acid (Figure 6). The RhB degradation kinetic was tracked by UV-Vis spectroscopy, adopting the main absorption band with a maximum around 550 nm as a reference. Furthermore, the decomposition of stearic acid was monitored using the CH_2_ stretching mode of FTIR.

**Figure 7:**
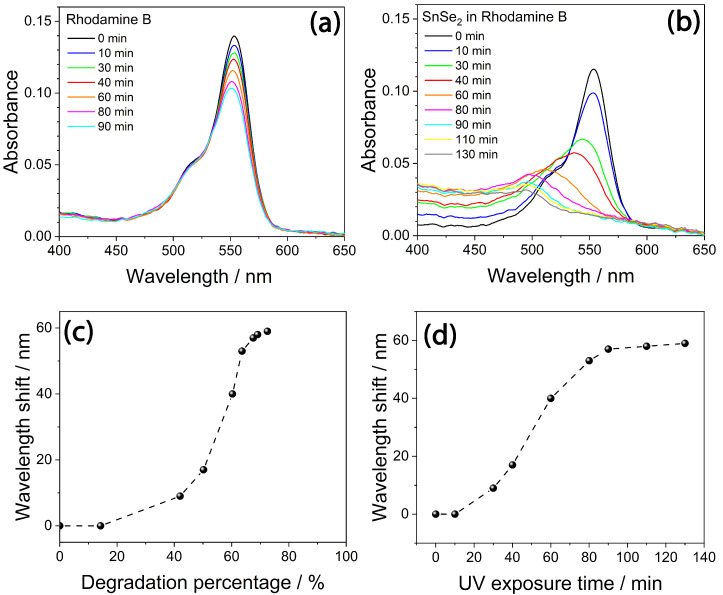
(**a**) UV–Vis absorption spectra of an aqueous solution of RhB (2.5 × 10^−6^ mol L^−1^) as a function of UV (365 nm) exposure time. (**b**) UV-Vis absorption spectra of the aqueous RhB solution containing dispersed SnSe_2_ flakes (0.07 g L^−1^) as a function of UV exposure time. (**c**,**d**) Percentage absorbance decrease and wavelength shift as a function of the UV exposure time. The data were taken from (**b**), using the maxima at 560 nm as reference for the calculation of the percentage decrease and wavelength shift. The line is a guide for the eyes.

The photocatalytic property of bare SnSe_2_ was verified before testing that of the m-TiO_2_-SnSe_2_ nanocomposite films. As already mentioned, nanostructured SnSe_2_ was rarely studied as a photocatalyst because of the fast electrons–holes recombination [47,48]. As a control, an aqueous solution of RhB was exposed to UV radiation for increasing periods of time. The results were compared to those obtained using another aqueous RhB solution, which also contained the SnSe_2_ flakes (Figure 7a,b).

Rhodamine B is a xanthene dye that is characterized by a strong absorption in the visible range, with a band around 550 nm [49] attributed to π → π* transitions. We used a 2.5 × 10^−6^ M concentration tabsorption spectra of an aqueous solution of RhBo avoid dimerization of the dye, which has a strong tendency to aggregate with the increase of the concentration, whereas monomers and dimers degrade at a different rate [50].

**Figure 8:**
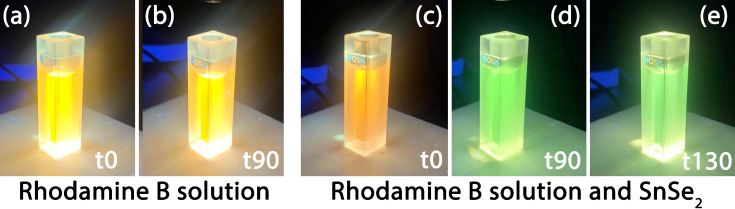
Optical images of RhB aqueous solutions (**a**,**b**) and RhB solution containing dispersed SnSe_2_ flakes (**c**–**e**) at different UV illumination times, t0 (before exposition), t90 (90 min), and t130 (130 min).

The absorption spectra show that only the monomeric form of Rhodamine B is present in solution (Figure 7a); RhB dimers have a strong absorption peaking around 530 nm that is not detected. The spectra in the 450–650 nm range are characterized by a broad and intense absorption band with a maximum around 550 nm, due to the delocalization, which involves the amines groups of the dye, and a vibronic shoulder peaking around 510 nm. The RhB control solution shows about 15% degradation after exposure to UV light for 90 min, in agreement with the photostability reported in the literature. The 550 nm absorbance measured in the RhB aqueous solution containing dispersed SnSe_2_ flakes decreased by 73% after 90 min of exposure, demonstrating that SnSe_2_ has a significant role in the photocatalytic activity (Figure 7c). At the same time, the maximum of the spectrum gradually shifts to around 500 nm (60 nm of blueshift) when the exposure time is longer than 90 min. (Figure 7d). The solution gradually turns from intense orange to green (Figure 8). The direct observation of the color change in the RhB aqueous solutions (Figure 8a,b) shows no differences after 90 min of exposure, confirming that RhB is not significantly degraded by UV in this exposure time. On the other hand, the color changes in the presence of bidimensional SnSe_2_ flakes indicate that the dye was chemically modified by the irradiation process (Figure 8c–e).

The decrease in intensity and the blueshift of the absorption maximum with the irradiation time indicate that RhB degradation occurs via de-ethylation [51,52]. As previously reported, the gradual blueshift (Figure 7b) suggests that the de-ethylation process activated by SnSe_2_ occurs stepwise, with the ethyl groups of RhB being removed in different stages [53]. When all the four ethyl groups have been removed from the dye, the molecule has the physical properties and chemical structure of Rhodamine 110 (Figure 6b). The final green color appearance of the dye suggests that an almost complete conversion of RhB into Rhodamine 110 has been achieved [54]. It has been observed that this peculiar photoactivated reaction is mostly related to a surface process which occurs when RhB is absorbed on the photocatalyst and allows radical water species (especially ^•^OH) to directly attack the ethyl groups of the dye structure. On the contrary, when the RhB is not absorbed on the photocatalyst, oxidative degradation of the dye chromophore prevails in solution, causing a general decrease of the absorbance, with no significant blueshift of the main absorption band [48]. Hence, the effective UV-photodegradation of RhB can be explained by the strong affinity between the SnSe_2_ flakes and the dye molecule.

**Figure 9:**
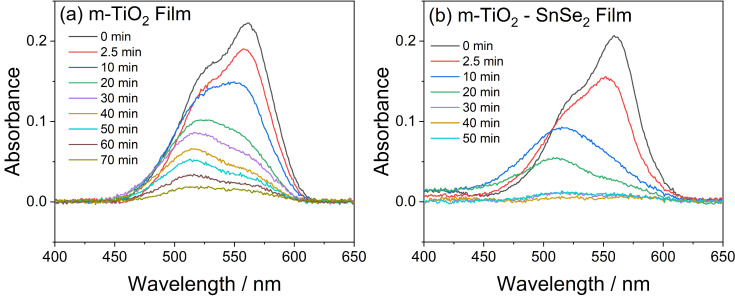
UV-Vis spectra of Rhodamine B deposited on m-TiO_2_ (**a**) and m-TiO_2_-SnSe_2_ (**b**) films as a function of UV exposure time.

Rhodamine B [55] has also been used as a test molecule to demonstrate the photocatalytic efficiency of the loaded film in comparison to the unloaded titania film. In both m-TiO_2_ and m-TiO_2_-SnSe_2_ systems, we observe that the RhB absorbance decreases and blueshifts as a function of the UV exposure time (Figure 9). On the nanocomposite film, the dye absorption band completely disappears after 30 min of irradiation; however, on the m-TiO_2_ film, a faint absorbance is detectable even after 70 min of UV exposure.

**Figure 10:**
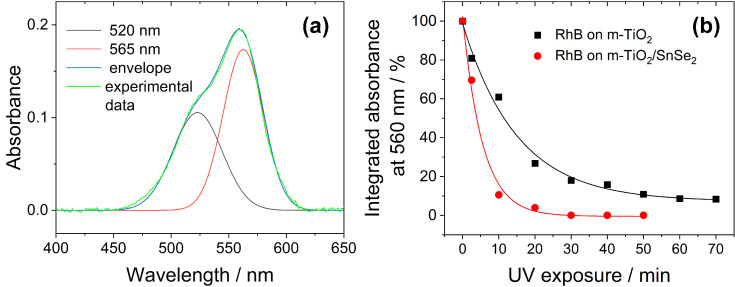
(**a**) Optical absorption spectrum of RhB deposited on m-TiO_2_ film and curve fit with 2 Gaussian functions. (**b**) Decrease of the integrated absorbance calculated from the Gaussian function peaked at 565 from the spectra of RhB deposited on m-TiO_2_ and m-TiO_2_-SnSe_2_ films (black squares and red dots, respectively). The lines depict the exponential decay fit of the experimental data.

To achieve a more detailed analysis of the photochemical changes occurring to the RhB deposited on the samples and UV-irradiated, we fitted the absorption in the 400–650 nm region, with two Gaussian functions taking into consideration two main contributions: one centered on 520 nm and the other with a maximum around 565 nm. (Figure 10a).

The contribution at shorter wavelengths is attributed both to the vibronic shoulder of the Rhodamine B and the de-ethylated rhodamine forms that show a blue-shifted main absorption, as seen before. The contribution at 565 nm, on the other hand, is unambiguously correlated with the electronic transition of not-photodegraded RhB and can thus be monitored to determine a more accurate photodegradation kinetic. Figure 10b is a plot of the integrated absorbance of the Gaussian function centered at 565 nm vs. the irradiation time. To estimate the photocatalytic efficiency, we used a decay law which follows pseudo-first-order kinetics [56,57,58]:

I(t) = I_0_·e^−kt^ (1)

where k is the degradation rate of pollutant molecules. The k-value of loaded films is 3 times higher than that of the unloaded sample (~0.182 min^−1^ vs. ~0.066 min^−1^ for m-TiO_2_-SnSe_2_ and m-TiO_2_, respectively). These results suggest that the heterostructure between SnSe_2_ and the nanocrystalline anatase titania increases the dye photodegradation.

**Figure 11:**
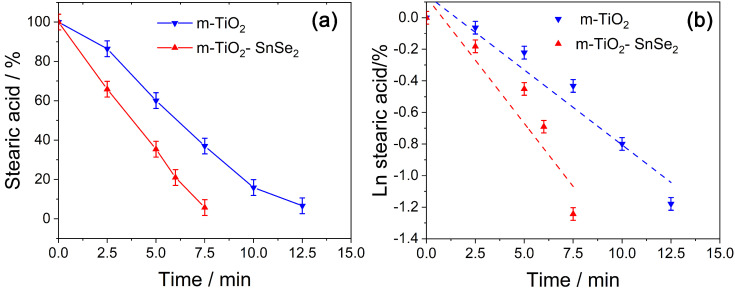
(**a**) Degradation rate (lines are a guide for eyes) obtained by measuring the decrease in intensity of the 2945 cm^−1^ C-H_2_ stretching mode and (**b**) decay law (dashed lines represent the decay fit) of stearic acid deposited on the different samples.

Notably, the UV-Vis spectroscopy monitoring of RhB photodegradation does not effectively offer information on the total removal of the organic molecule but rather on the chemical changes occurring on the dye molecule as a result of photodegradation. To acquire a better understanding of the self-cleaning properties of the nanocomposite films, stearic acid solution (5.0 g L^−1^) was spin-coated onto the films as a model pollutant to be decomposed by UV irradiation. FTIR spectroscopy was used to evaluate the photocatalytic degradation of stearic acid by monitoring the intensity of the -CH_2_ stretching mode, as shown in Figure 11a,b for m-TiO_2_ and m-TiO_2_-SnSe_2_, respectively.

Figure 11 displays the degradation rate of stearic acid (stearic acid/%), which is evaluated by the following:

Stearic acid/% = I_t_/I_0_·100 (2)

where I_t_/I_0_ is the ratio between the infrared absorption intensity at t irradiation time, I_t_, and the absorption intensity before exposure, I_0_.

m-TiO_2_ (blue line in Figure 11a) is able to remove more than 80% of stearic acid from the surface after 10 min of exposure. Nevertheless, the m-TiO_2_-SnSe_2_ film degrades the pollutant at a quicker rate, achieving a 90% degradation after only 7.5 min. The dashed straight lines in Figure 11b represent the decay law that follows a pseudo-first-order kinetics (1).

The calculated k-value for m-TiO_2_ film is ~0.092 min^−1^, while for m-TiO_2_-SnSe_2_ film, it is ~0.160 min^−1^. This confirms that the SnSe_2_ flakes enhance the photoactivity of titania mesoporous film by increasing the degradation rate more than 70% with only 7% of loading (see Table 1).

The experiments with the stearic acid are complementary to those with the organic dye. The measurement obtained by FTIR on stearic acid provides a quantitative indication of the organic removal from the surface, simulating a real self-cleaning process. Moreover, the absorbance variations of Rhodamine B provide evidence for the hypothesis that interactions between the dye and the nanocomposite films’ surfaces are critical for boosting photocatalysis.

The photocatalytic activity shown by the m-TiO_2_-SnSe_2_ film can be applied to produce anti-fingerprint coatings. This function has been evaluated by immersing the finger in a stearic acid solution, pressing the finger to the substrate, and observing its removal under exposure to sunshine or ultraviolet radiation [59,60]. We selected this testing method to obtain a direct comparison and more reproducible findings in contrast to direct human fingerprints, whose chemical composition is more challenging to duplicate.

**Figure 12:**
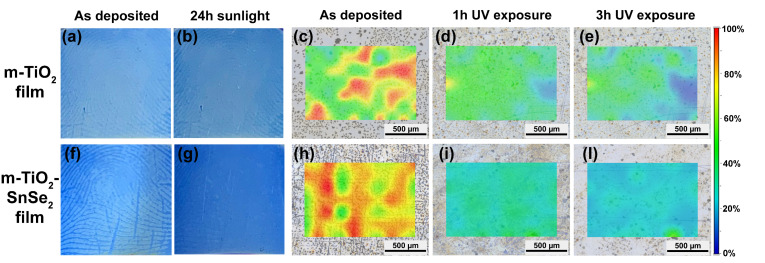
Photographs of human fingerprints on m-TiO_2_ (**a**,**b**) and m-TiO_2_-SnSe_2_ (**f**,**g**) films before and after exposure to sunlight in air for 24 h. Infrared images of a human fingerprint deposited on m-TiO_2_ (**c**–**e**) and m-TiO_2_-SnSe_2_ (**h**–**l**) films. The images were taken by integrating the area of the -CH_2_ stretching bands of stearic acid. The fingerprint was produced by immersing the finger in a stearic acid solution. The infrared images were detected at different times: as deposited, after 1 h and after 3 h of UV exposure, respectively. The color scale bar shows the intensity scale in false colors; the red and blue colors represent the highest and the lowest absorbance of the infrared signal, respectively.

Figure 12a,b,f,g display some representative images of m-TiO_2_ and m-TiO_2_-SnSe_2_ films before and after fingerprint degradation under ambient conditions (sunlight). The observation of a latent fingerprint on the surface of the m-TiO_2_-SnSe_2_ film (Figure 12g), as opposed to the clear residual pattern detected in the m-TiO_2_ film (Figure 12b), indicated that the fingerprint was fully degraded after 24 h. The test validates the nanocomposite’s improved photocatalytic degradation performance compared to that of the bare m-TiO_2_ film.

Figure 12c–e,h– l show the infrared images performed to evaluate the degradation of a fingerprint deposited on m-TiO_2_ and m-TiO_2_-SnSe_2_ films at different UV exposure times (Figure 12). The infrared images were taken by integrating the area of the -CH_2_ stretching bands of stearic acid. After 1 h of UV exposure, the infrared images showed that the m-TiO_2_-SnSe_2_ film almost completely cancelled the fingerprint that is still detected on the m-TiO_2_ film (Figure 12d).

**Figure 13:**
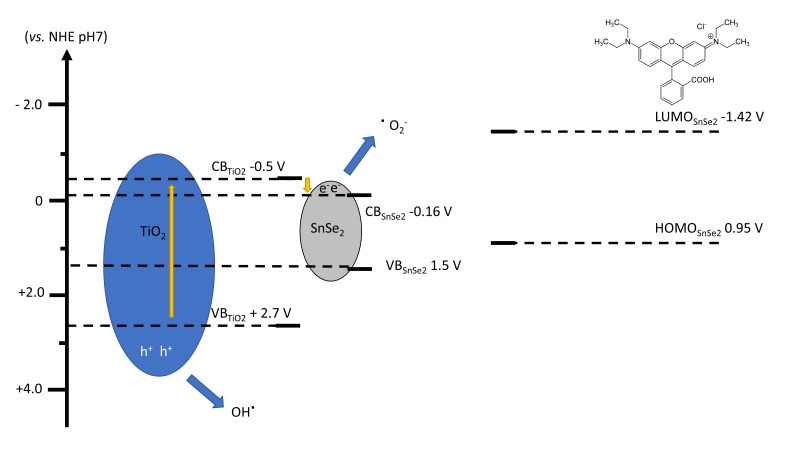
Hypothesis of the Rhodamine B degradation mechanism in presence of SnSe_2_ nanoflakes under UV-Visible light. NHE, normal nitrogen electrode; CB, conduction band; VB, valence band; LUMO, lowest unoccupied molecular orbital; HOMO, highest unoccupied molecular orbital.

Figure 13 outlines the possible mechanism of photocatalysis occurring in the nanocomposite films [61]. When the UV light is absorbed by the titania matrix, the valence band (VB) electrons are excited to the conduction band (CB) and then transferred to that of SnSe_2_. The UV light also interacts with SnSe_2_ nanoflakes, which have a broad absorption up to the infrared range. However, we observed that a 7% SnSe_2_ loading has no effect on the films’ absorbance. This implies that the primary impact of SnSe_2_ is not to boost the light-harvesting efficiency but rather to decrease the total hole and electron recombination rate in the heterojunction. As a consequence of the light absorption, photogenerated electrons (e^-^) and holes (h^+^) on the surface of the photo-catalyst generate free radicals such as hydroxyl radicals (•OH) and superoxide radical anions (•O_2_^−^). The •OH radicals are produced by the reaction between e^−^/h^+^ pairs and OH^−^/H_2_O in the VB. At the same time, the excited electrons in the CB react with the dissolved oxygen in the solution to form •O_2_^−^, which reacts with H^+^ to form other hydroxyl radicals (•OH and O_2_•^-^). These highly active hydroxyl radical species can oxidize the organic molecules, hence promoting the self-cleaning effect [62].

## 4. Conclusions

The photophysical properties of bidimensional SnSe_2_ make it an excellent candidate for the development of photoactive heterostructures in combination with titania. Bidimensional SnSe_2_ flakes in solution have a high photocatalytic activity towards xanthene dyes such as Rhodamine B. The flakes can be directly added to a titania precursor sol to fabricate nanocomposite mesoporous ordered films via one-pot self-assembly without disruption of the mesophase organization. Remarkably, the optical quality of the films is not affected by the SnSe_2_ addition, and the difference in the film transmittance is negligible, ensuring the transparency of the coatings in the whole visible range. The nanocomposite films show a higher photocatalytic activity in comparison to mesoporous titania films. The cooperative effect of the two photocatalysts reduces the recombination rate of the photogenerated electron/hole couple. The effective degradation of human fingerprints on the film surface confirms that the specifications for anti-fingerprint coatings are met excellently by titania mesoporous nanocomposite films.

## Figures and Tables

**Table 1:** Thickness, porosity and SnSe_2_ loading measured by ellipsometry for three m-TiO_2_ and m-TiO_2_-SnSe_2_ films (Samples 1, 2, and 3).

	m-TiO_2_		m-TiO_2_-SnSe_2_		
	Thickness (nm)	Porosity (%)	Thickness (nm)	Porosity (%)	SnSe_2_ (%)
Sample 1	121.3 ± 0.3	37.1 ± 0.1	138.7 ± 0.3	27.5 ± 0.1	6.5 ± 0.1
Sample 2	117.9 ± 0.6	38.8 ± 0.2	142.0 ± 0.3	26.4 ± 0.1	8.1 ± 0.1
Sample 3	124.1 ± 0.9	39.5 ± 0.2	139.9 ± 0.4	27.3 ± 0.2	8.9 ± 0.1

## Data Availability

Original data are available upon request to the corresponding author.

## Supplementary Materials

The following supporting information can be downloaded at https://www.mdpi.com/article/10.3390/nano13081406/s1, Figure S1. Calculation of the band gap from UV-Vis spectra; Figure S2. EDS analysis of SnSe_2_ flakes into m-TiO_2_ film; Figure S3. SEM characterization of the m-TiO_2_-SnSe_2_ samples; Figure S4: N2 gas adsorption isotherm of the m-TiO_2_-SnSe_2_ nanocomposite; Figure S5. Contact angle images. References [63,64] is cited in the supplementary materials.

## Abbreviations

m-TiO_2_, mesoporous ordered titania films; m-TiO_2_-SnSe_2_, mesoporous ordered titania films containing dispersed SnSe_2_ nanomaterials; RhB, Rhodamine B; LPE, liquid phase exfoliation; NHE, normal nitrogen electrode; CB, conduction band; VB, valence band; LUMO, lowest unoccupied molecular orbital; HOMO, highest unoccupied molecular orbital.
