# Supplementary material for: Bidimensional SnSe2—Mesoporous Ordered Titania Heterostructures for Photocatalytically Activated Anti-Fingerprint Optically Transparent Layers
Source: Nanomaterials (Basel). 2023 Apr 19;13(8):1406. doi: 10.3390/nano13081406 (PMC10143690; doi:10.3390/nano13081406)
Supplement: Supplementary file 1 [file nanomaterials-13-01406-s001.zip › nanomaterials-2346276-supplementary.pdf]

SUPPORTING INFORMATION

Bidimensional $SnSe_2$ – mesoporous ordered titania heterostructures for photocatalytically activated anti-fingerprint optically transparent layers

Jessica De Santis[1,2], Valentina Paolucci[1,2], Carlo Cantalini[1,2*], Luigi Stagi[3], Davide Carboni[2,3], Luca Malfatti[2,3], Plinio Innocenzi[2,3,4*]

[1] Department of Industrial and Information Engineering and Economics, 67100 L'Aquila, Italy; jessica.desantis@graduate.univaq.it (J.D.S.); valentina.paolucci2@univaq.it (V.P.); carlo.cantalini@univaq.it (C.C.)
[2] National Interuniversity Consortium of Materials Science and Technology (INSTM), 50121 Florence, Italy;
[3] Laboratory of Materials Science and Nanotechnology (LMNT), Department of Biomedical Sciences, CR-INSTM, University of Sassari, 07100 Sassari, Italy; lstagi@uniss.it (L.S.); lucamalfatti@uniss.it (L.M.); plinio@uniss.it (P.I.); dcarboni@uniss.it (D.C.)
[4] College of Science, Department of Chemistry. United Arab Emirates University. 15551 Al Ain. United Arab Emirates

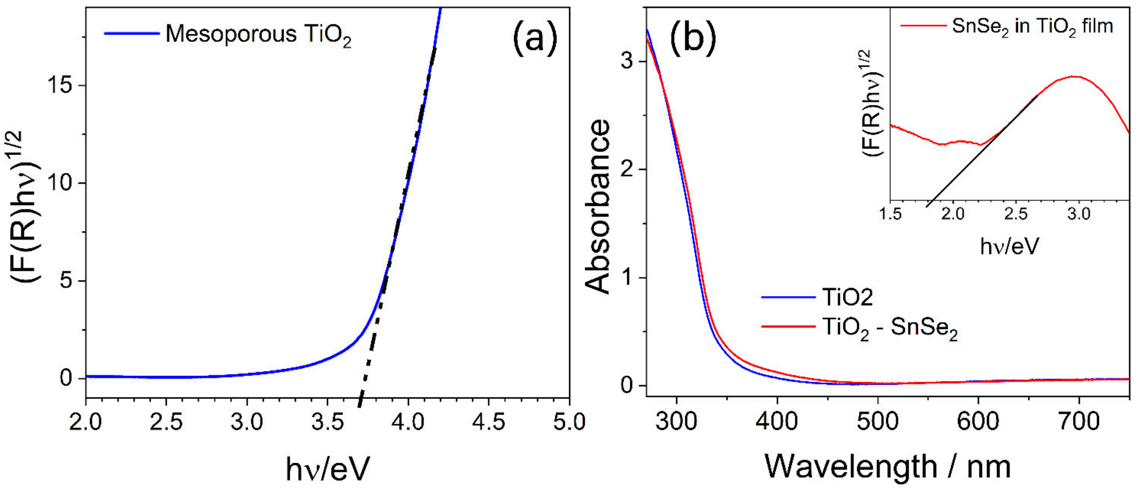

Figure S1. Calculation of the band gap from UV-Vis spectra. (a) Tauc plot of the m-TiO_2 mesoporous film. b) UV-Vis spectra of m-TiO_2 and m-TiO_2-$SnSe_2$ films. Inset: Tauc plot of exfoliated $SnSe_2$.

Figure S1a shows the indirect optical band gap of a mesoporous TiO_2 film deposited on a silica glass substrate, estimated by plotting F(R)hν vs hν curves. The measured band gap is 3.7 eV, in line with previous reports [63].

Figure S1b shows the UV-Vis absorption spectra of m-TiO_2 (blue line) and m-TiO_2 - $SnSe_2$ (red line) films on a silica glass substrate. No significant differences have been detected. Additionally, the inset in **Figure S1b**, displays the Tauc plot of $SnSe_2$; the calculated optical band gap is ~1.8 eV, which is line with the value of exfoliated $SnSe_2$ (see microstructural characterization in the manuscript) and previous reports [31,64].

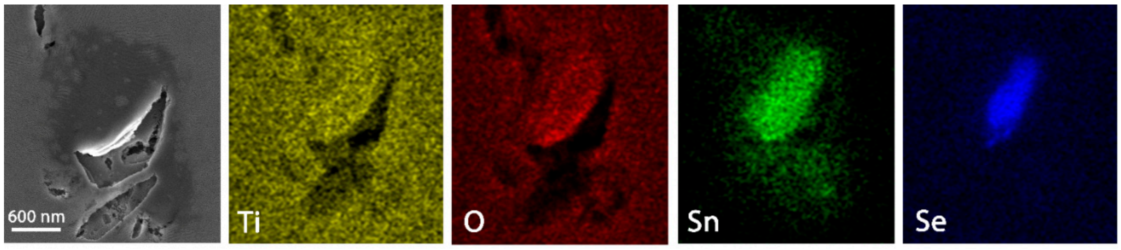

Figure S2. EDS analysis of $SnSe_2$ flakes into m-TiO_2 film

Figure S2 shows the EDS mapping of the $SnSe_2$ flake in the m-TiO_2 film. The background is the titania films and the corresponding signals from Ti and O appear homogeneously distributed. In the small crack in the film an increase of the signal from the Sn and Se is observed. This indicates that the flake are incorporated inside the films.

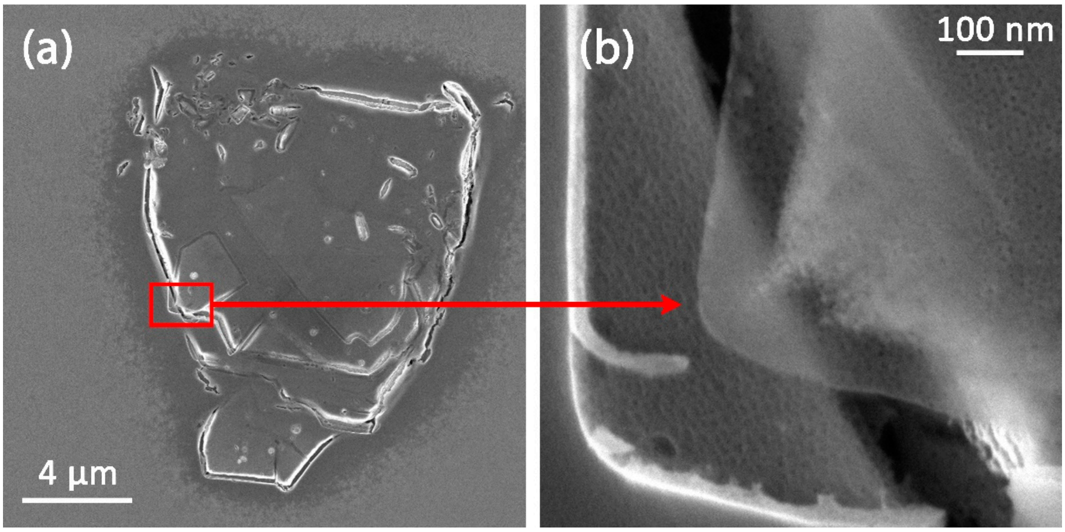

Figure S3. SEM characterization of the m-TiO_2-$SnSe_2$ samples. (a) Analysis of an $SnSe_2$ flake, (b) magnification of a single sheet where can be observed the TiO_2 mesoporous texture.

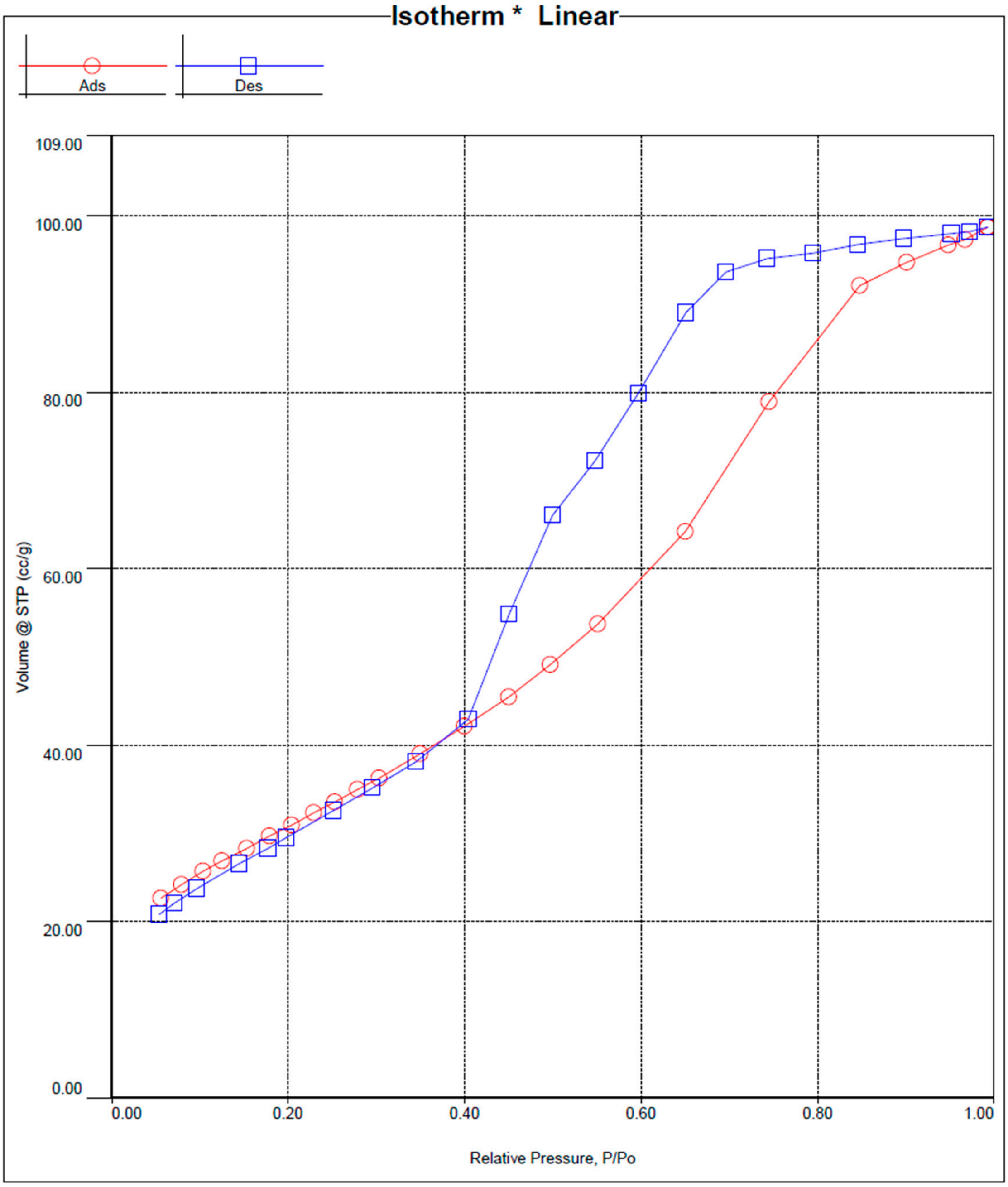

Figure S4. N_2 gas adsorption isotherm of the m-TiO_2-$SnSe_2$ nanocomposite.

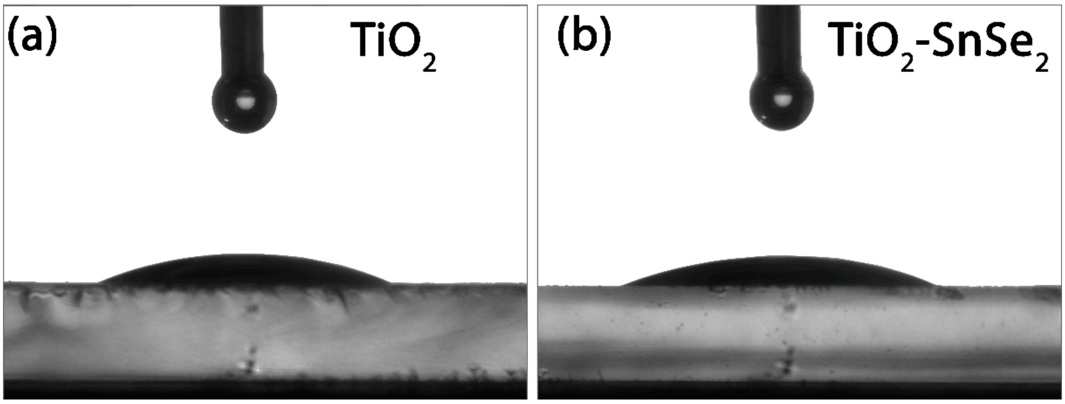

Figure S5. Contact angle images obtained using a water droplet deposited on (a) m-TiO_2 and (b) m-TiO_2-$SnSe_2$ films.

Figure S5 shows the contact angle measurements used to investigate the surface wettability of the films. Both films deposited on a glass substrate show the same hydrophilic properties, with contact angle of 23.25 and 22.8° for undoped and doped m-TiO_2, respectively. Regardless the presence of $SnSe_2$ nanosheets, the contact angle remains the same, so it means that the film does not change the hydrophilic properties.

References

31. Gonzalez, J.M.; Oleynik, I.I. Layer-Dependent Properties of SnS2 and SnSe2 Two-Dimensional Materials. *Phys. Rev. B* **2016**, *94*, 1–10. https://doi.org/10.1103/PhysRevB.94.125443.
63. Aadim, K.A.; Haneen, K.; Hadi, Q.M. Effect of Annealing Temperature on the Optical Properties of TiO2 Thin Films Prepared by Pulse Laser Deposition. *International Letters of Chemistry, Physics and Astronomy.* **2015**, *56*, 63–70. 10.18052/www.scipress.com/ILCPA.56.63.
64. Rahman, A.; Kim, H.J.; Noor-A-Alam, M.; Shin, Y.H. A theoretical study on tuning band gaps of monolayer and bilayer SnS_2 and $SnSe_2$ under external stimuli. *Curr. Appl. Phys.* **2019**, *19*, 709. https://doi.org/10.1016/j.cap.2019.03.008.
